# Functional investigation of *SLC1A2* variants associated with epilepsy

**DOI:** 10.1038/s41419-022-05457-6

**Published:** 2022-12-21

**Authors:** Qi Qu, Wenlong Zhang, Ji Wang, Dongmei Mai, Siqiang Ren, Shaogang Qu, Yunlong Zhang

**Affiliations:** 1 Department of Neurology, Nanfang Hospital, Southern Medical University, Guangzhou, Guangdong 510515 China; 2 Department of Neurobiology, School of Basic Medical Sciences, Southern Medical University, Guangzhou, Guangdong 510515 China; 3 Guangdong-Hong Kong-Macao Greater Bay Area Center for Brain Science and Brain-Inspired Intelligence, Guangzhou, Guangdong 510515 China; 4 Key Laboratory of Mental Health of the Ministry of Education, Southern Medical University, Guangzhou, Guangdong 510515 China; 5 Department of Neurology, The First Affiliated Hospital of Guangzhou Medical University, Guangzhou, 510120 China; 6 Key Laboratory of Neuroscience, School of Basic Medical Sciences, Guangzhou Medical University, Guangzhou, 511436 China

**Keywords:** Transporters in the nervous system, Epilepsy

## Abstract

Epilepsy is a common neurological disorder and glutamate excitotoxicity plays a key role in epileptic pathogenesis. Astrocytic glutamate transporter GLT-1 is responsible for preventing excitotoxicity via clearing extracellular accumulated glutamate. Previously, three variants (G82R, L85P, and P289R) in *SLC1A2* (encoding GLT-1) have been clinically reported to be associated with epilepsy. However, the functional validation and underlying mechanism of these GLT-1 variants in epilepsy remain undetermined. In this study, we reported that these disease-linked mutants significantly decrease glutamate uptake, cell membrane expression of the glutamate transporter, and glutamate-elicited current. Additionally, we found that these variants may disturbed stromal-interacting molecule 1 (STIM1)/Orai1-mediated store-operated Ca^2+^ entry (SOCE) machinery in the endoplasmic reticulum (ER), in which GLT-1 may be a new partner of SOCE. Furthermore, knock-in mice with disease-associated variants showed a hyperactive phenotype accompanied by reduced glutamate transporter expression. Therefore, GLT-1 is a promising and reliable therapeutic target for epilepsy interventions.

## Introduction

Epilepsy is a common chronic neurological disease characterized by an enduring predisposition to epileptic seizures [1, 2]. Seizures include focal, generalized, combined generalized and focal, and unknown types [3]. Epilepsy affects approximately 65 million people worldwide and is associated with an increased incidence of injury and mortality [3]. Genetic and environmental factors, infections, neuroinflammation, and gut microbiome disequilibrium are involved in epilepsy pathogenesis and outcome [4–8]. Importantly, genetic risk factors account for 35% of the epileptic morbidity rate [9], and several risk genes, such as *GABRB3*, *GABRA1*, *SCN1A*, *SCN2A*, *GRIN2A*, *ASH1L*, and *CNKSR2*, have been investigated [10–16]. Among these risk genes, *SLC1A2* (which encodes for the excitatory amino acid transporter 2, EAAT2, also called GLT-1) variants, i.e., Gly82Arg, Leu85Pro, and Pro289Arg, are clinically associated with epilepsy [17–19]. In addition, loss of *EAAT2A* induces neuronal hyperexcitability and recurrent epileptic seizures in zebrafish [20]. Genetic and pharmacological upregulation of GLT-1, and inhibition of GLT-1 degradation, hinder seizure activity and prevent cognitive impairment in epileptic animal models [21–23]. These data suggest that GLT-1 plays a critical role in epilepsy pathogenesis and treatment.

Glutamate excitotoxicity plays a well-established role in the pathophysiology of epilepsy [24, 25]. Increased extracellular glutamate levels were found in epileptogenic regions of patients with epilepsy [26, 27]. The resulted neuronal death due to glutamate excitotoxicity thus became a cause and consequence of epileptic seizures [28, 29]. Epileptic risk genes also mediate seizure via glutamate excitotoxicity, such as *ASH1L* deficiency-induced severe seizures through attenuating GABAergic inhibition and enhancing glutamatergic transmission, which resulting in elevated pyramidal neuronal excitability [15]. Notably, glutamate transporters are responsible for removing glutamate from the extracellular space following glutamate release into the synaptic cleft in the central nervous system (CNS). Astrocytic GLT-1 is a key contributor to maintaining low levels of extracellular glutamate and is responsible for 90% of glutamate clearance from the synaptic clefts in the CNS [30]. Glutamate transporters contain six transmembrane domain segments, consisting of transmembrane domains 3 (TM3), TM6-8, two reentrant helical hairpin loops 1 (HP1), HP2, and trimerization domain, including TM2, 4, and 5 [31, 32]. The glutamate transport process is driven by the cotransport of three sodium ions and one proton ion, and the counter-transport of one potassium ion using the energy generated by Na^+^/K^+^-ATPase [33]. Previously, several groups, including ours, have investigated the relationship between the critical domains or sites during the conformational shift of glutamate transporters [31, 34–37]. Furthermore, we also revealed the crucial role of glutamate transporters in neurodegenerative diseases, such as Parkinson’s disease (PD) and Alzheimer’s disease (AD) [38–40]. The trimeric interface of glutamate transporters involves TM2, 4, and 5, and remains unchanged during transport [41]. Intriguingly, these three *de novo* mutations are localized within TM2 and TM5, and it is unclear whether these mutations affect the transport cascade of GLT-1. Although *SLC1A2* null mice manifest seizures [42], it is unclear whether locus mutations of *SLC1A2* mice lead to epilepsy-associated phenotype.

In this study, we reported that disease-linked mutations (G82R, L85P, and P289R) significantly decreased the glutamate uptake, cell membrane expression of glutamate transporters, and glutamate-mediated current. We also showed that these variants may disturb the function of stromal-interacting molecule 1 (STIM1)/Orai1-mediated store-operated Ca^2+^ entry (SOCE) in the endoplasmic reticulum (ER). To further explore the role of glutamate transporters in epilepsy, we established knock-in (KI) mice with disease-associated variants and found them to have a hyperactive behavior accompanied by reduced glutamate transporter expression. Our data suggest that microglial activation may act in coordination with glutamate excitotoxicity to induce neuronal hyperexcitability in epilepsy.

## Results

### Epileptic variants decrease the expression and function of glutamate transporter GLT-1

**Fig. 1:**
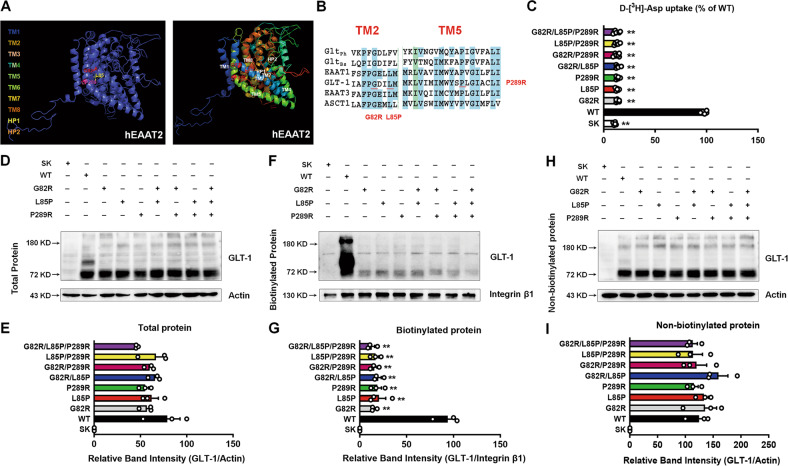
Epileptic variants decrease the expression and function of GLT-1. **A** Topological location of Gly-82, Leu-85, and Pro-289 residues in human EAAT2. **B** Comparison of the amino acid sequences of epileptic variants (G82R, L85P, and P289R) between Glt_ph_ and EAATs. Regions of high homology of Glt_Ph_ and EAATs are highlighted in blue. **C** D-[^3^H]-Asp uptake activity after transfection with different plasmids (SK, WT, G82R, L85P, P289R, G82R/L85P, G82R/P289R, L85P/P289R, and G82R/L85P/P289R) were determined. *n* = 6 per group. The representative blots and quantification of total proteins (**D**, **E**), biotinylated membrane proteins (**F**, **G**), and non-biotinylated proteins (**H**, **I**) of GLT-1 were measured by Western blot. *n* = 3 per group. Results are expressed as mean ± SD. ^**^P < 0.01 vs. WT. Statistical significance was determined using one-way ANOVA and Tukey’s test for post hoc comparisons.

To date, three *SLC1A2* variants (Gly82Arg, Leu85Pro, and Pro289Arg) have been reported to be associated with epilepsy [17–19]; we identified their topological location and the different domains of human EAAT2 (Fig. 1A). The Gly-82 and Leu-85 residues reside in the TM2 domain and the Pro-289 residue resides in the TM5 domain of GLT-1, and these three residues are highly conserved between eukaryotic glutamate transporters EAATs and Glt_ph_, a prokaryotic homolog of EAATs (Fig. 1B). To address the effects of these three mutants on the transport process and determine whether they induce relative motion between TM2 and TM5, we constructed single disease mutants (G82R, L85P, and P289R), double mutants (G82R/L85P, G82R/P289R, and L85P/P289R), and triple mutants (G82R/L85P/P289R). All mutants significantly decreased the transport activity (~90%) using radiolabeled D-aspartate, the analogous transport substrate as glutamate (Fig. 1C). Glutamate transporters perform their transport function due to their precise expressions on the cell membrane [38]. The mutants significantly reduced the biotinylated membrane expression of GLT-1 but did not have any obvious effect on the total and non-biotinylated GLT-1 expression levels (Fig. 1D–I), consistent with their effects on the transport activity.

**Fig. 2:**
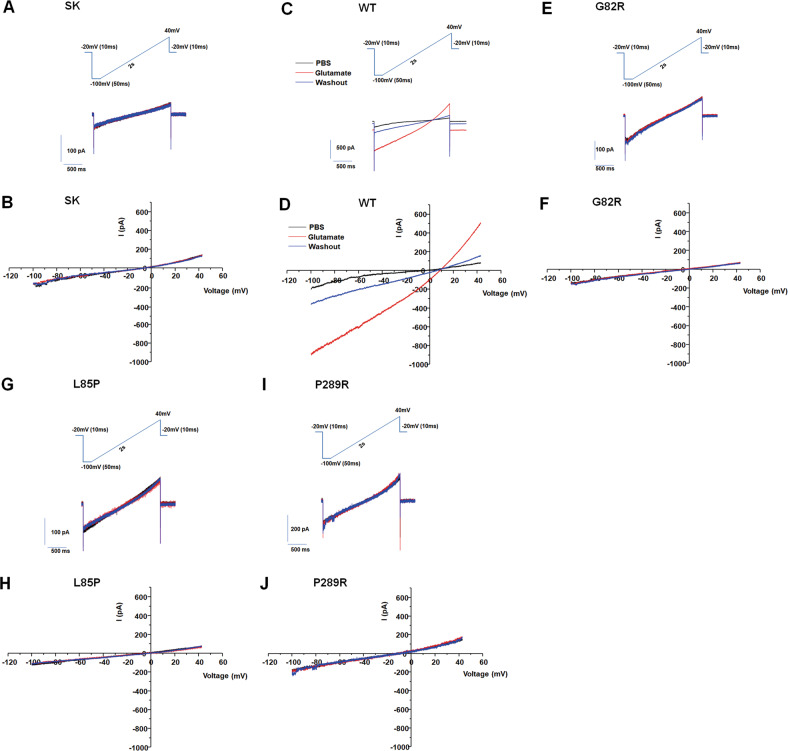
Epileptic variants inhibited glutamate-elicited currents. Glutamate-elicited inward currents after transfection with different plasmids, including SK (**A**, **B**), WT (**C**, **D**), G82R (**E**, **F**), L85P (**G**, **H**), and P289R (**I**, **J**), were evaluated by electrophysiology. Representative traces of PBS, glutamate, and washout are presented in (**A**, **C**, **E**, **G**, and **I**).

**Fig. 3:**
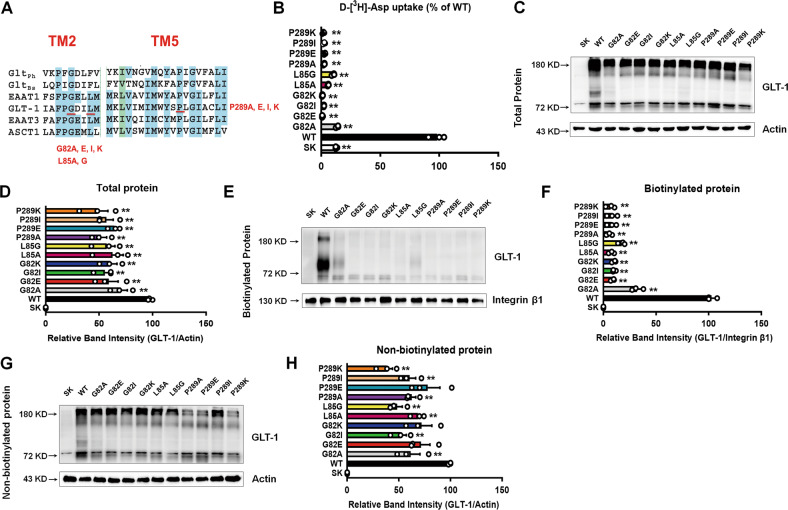
Epileptic variants did not affect the side-chain structure of GLT-1. **A** The three epileptic variants were mutated with amino acids with similar properties as the original amino acids (G82A, G82E, G82I, G82K; L85A, L85G; P289A, P289E, P289I, P289K). **B** After transfection with different plasmids, D-[^3^H]-Asp uptake activity was determined. *n* = 3 per group. The representative blots and quantification of total proteins (**C**, **D**), biotinylated membrane proteins (**E**, **F**), and non-biotinylated proteins (**G**, **H**) of GLT-1 were measured by Western blot. *n* = 3 per group. Results are expressed as mean ± SD. ^**^P < 0.01 vs. WT. Statistical significance was determined using one-way ANOVA and Tukey’s tests for post hoc comparisons.

Because the glutamate transport process is accompanied by a coupled inward current mediated by sodium-coupled glutamate translocation, and an uncoupled current mediated by chloride ions [43, 44], we examined the effects of these disease-associated variants on the glutamate-elicited current. Glutamate supplementation induced an inward current after transfection of WT plasmid in HEK293 cells compared to transfection of SK (vector) plasmid (Fig. 2A–D). The G82R, L85P, and P289R variants significantly abolished the glutamate-elicited inward current (Fig. 2E–J), suggesting that they affected the function of glutamate transporters. To exclude the potential effects of disease-associated variants disturbing the side-chain structures of glutamate transporters, we further substituted these residues with other amino acids (Fig. 3A). G82, L85, and P289 were replaced with similar nonpolar hydrophobic amino acid alanine (G82A, L85A, and P289A), isoleucine (G82I and P289I), and glycine (L85G) [45]. However, the transport activity and cell surface protein level did not recover (Fig. 3B, E, and F). In addition, when G82 and P289 were substituted with the negatively charged glutamic acid (G82E and P289E) or positively charged lysine acid (G82K and P289K) [46], the function and protein level were still reduced (Fig. 3B, E, and F). These results suggest that the effects of the three variants on impaired transport activity were not related to the structure, charge, or polarity of the amino acid side chain. Furthermore, these mutants suppressed the total expression of GLT-1, particularly the majority of non-biotinylated GLT-1 (Fig. 3C, D, G, and H). These data suggest that the disease-associated variants may not affect the side-chain structures of glutamate transporters.

### Epileptic variants may affect STIM1/Orai1-mediated SOCE in ER

**Fig. 4:**
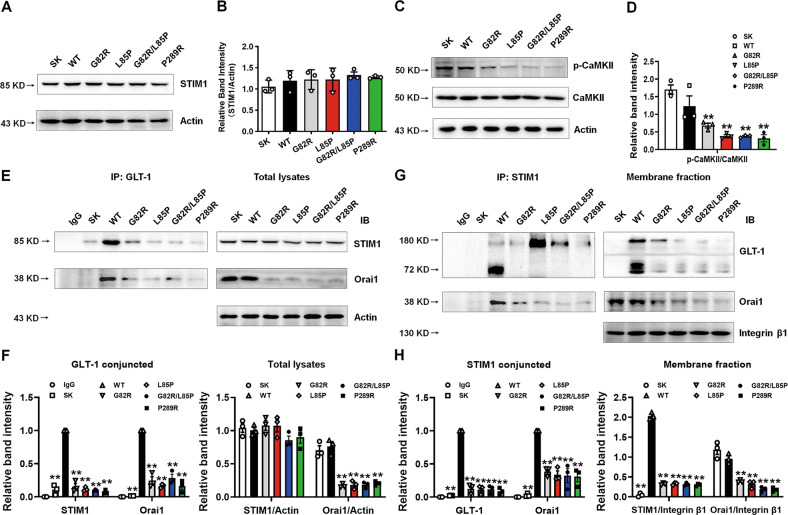
Epileptic variants affected the STIM1/Orai1 complex. **A**, **B** The representative blots and quantification of STIM1 after transfection with different plasmids (SK, WT, G82R, L85P, G82R/L85P, and P289R) in HeLa cells were determined by Western blot. *n* = 3 per group. **C**, **D** The representative blots and quantification of the phosphorylation of CaMKII and total CaMKII after transfection with different plasmids in HeLa cells were determined by Western blot. *n* = 3 per group. **E**, **F** The interaction between GLT-1 and STIM1 or Orai1 was determined by a Co-IP assay using GLT-1 as the capture antibody. **G**, **H** The interaction between GLT-1 and STIM1 or Orai1 was determined by a Co-IP assay using STIM1 as the capture antibody. *n* = 3 per group. Results are expressed as mean ± SD. ^**^P < 0.01 vs. WT. Statistical significance was determined using one-way ANOVA and Tukey’s tests for post hoc comparisons.

**Fig. 5:**
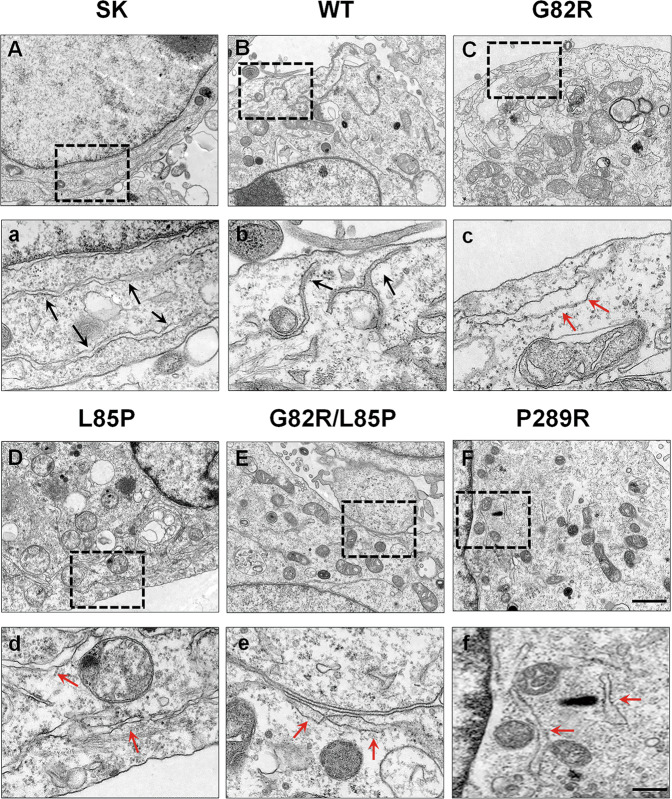
Disease-related variants altered the morphology of the endoplasmic reticulum (ER). After transfection with different plasmids (SK, WT, G82R, L85P, G82R/L85P, and P289R) in HeLa cells, the morphology of ER was observed using TEM (**A**–**F**). The epileptic variants induced ER swelling (as indicated by the red arrows in (**C**–**F**). Scale bar, 1 μm. Magnified images are shown in the column of (**a**–**f**). Scale bar, 200 nm.

Our previous work indicated that astrocytic knockdown of GLT-1 impairs the calcium signaling pathway [39]. In astrocytes, Ca^2+^ in the ER lumen is the major source of intracellular Ca^2+^, and SOCE links ER with plasmalemmal Ca^2+^ entry [47, 48]. The depletion of ER luminal Ca^2+^ triggers STIM, the ER-resident Ca^2+^ sensor, to interact with plasmalemmal SOCE channels, including both Orai and transient receptor potential (TRP) families. The activation of SOCE results in the influx of extracellular Ca^2+^ into the cytosol and subsequent refilling of ER stores [49]. Although we did not identify any effect of these mutants on the expression of STIM1 (Fig. 4A, B), they decreased the phosphorylation of CaMKII (Fig. 4C, D), which was reported to enhance SOCE activity [50]. Then, we examined the interaction of GLT-1 with STIM1, using GLT-1 as the capture antibody, and found that these epileptic variants decreased the interaction between STIM1 or Orai1 and GLT-1 (Fig. 4E, F). Consistently, using STIM1 as the capture antibody, we found that the epileptic variants inhibited the interaction between GLT-1 or Orai1 and STIM1 (Fig. 4G, H). Interestingly, these mutants decreased the total and membrane Orai1 expression (Fig. 4E–H). These data suggest that GLT-1 may be a new partner of SOCE and the epileptic variants of GLT-1 may reduce SOCE activity. We further verified our hypothesis using TEM (Fig. 5A–F) and found that the epileptic variants induced ER swelling (indicated by red arrows in Fig. 5C–F), which supports their effect on SOCE.

### Establishment and evaluation of G82R/L85P variant KI mice

**Fig. 6:**
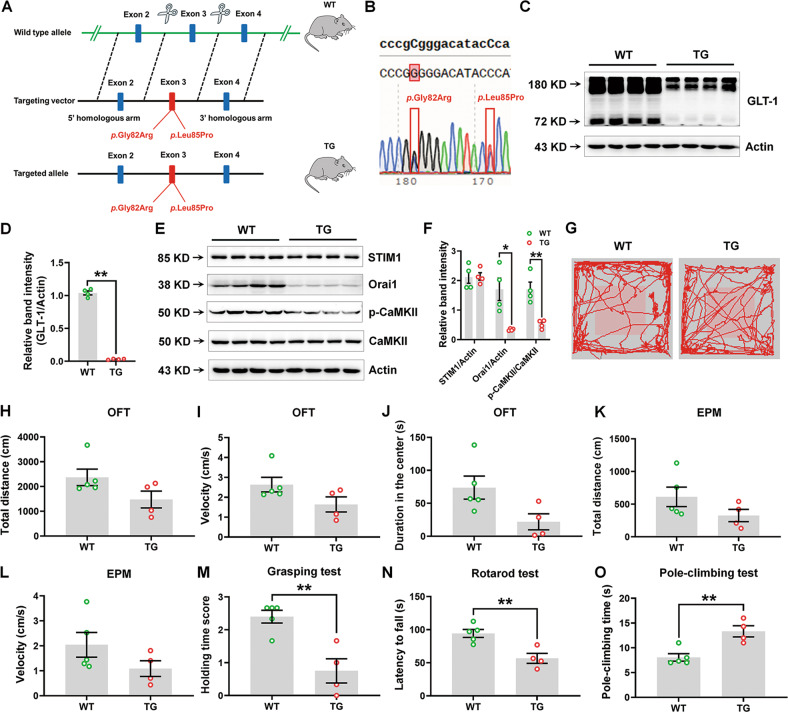
Establishment of G82R/L85P variant knock-in mice. **A**, **B** Strategy of establishing the G82R/L85P variant knock-in mice. **C**, **D** Representative blots and quantification of GLT-1 in the hippocampus of WT and TG mice were determined by Western blot. *n* = 4 per group. **E**, **F** The representative blots and quantification of STIM1, Orai1, phosphorylation of CaMKII, and total CaMKII in the hippocampus of WT and TG mice were determined by Western blot. *n* = 4 per group. **G** The representative trace mice traveled in the open field. The total traveled distance (**H**), movement speed (**I**) in the OFT, and duration in the center zone of open field (**J**) are shown. **K**, **L** The total traveled distance and movement speed in the EPM test. **M** The grasping test was used to examine the grip strength of mice. **N** The rotarod test was used to examine the motor coordination of mice. **O** The pole-climbing test was used to examine bradykinesia in mice. *n* = 5 for WT mice (four males and one female) and *n* = 4 for TG mice (three males and one female). Results are expressed as mean ± SD. ^**^P < 0.01, ^**^P < 0.05 vs. WT. Statistical significance was determined using Student’s *t* test.

Both Gly-82 and Leu-85 residues reside in the TM2 domain. In contrast to the previously held belief that TM2 is rigid during the transport process [41], we recently reported that TM2 of EAAT2 is critical for membrane-bound localization [51]. We established transgenic (TG) mice possessing the G82R/L85P variant (Fig. 6A, B). The GLT-1 expression was nearly abolished in the hippocampus of TG mice (Fig. 6C, D), which was consistent with our in vitro results. In addition, the expressions of Orai1 and phosphorylation of CaMKII, but not STIM1, were decreased in the hippocampus of TG mice (Fig. 6E, F), which was in line with the in vitro results that SOCE may be disturbed. Compared to WT mice, TG mice run across the cage rapidly, which may be due to hyperactivity (Supplementary Videos 1 and 2). However, the behavioral performance in the OFT, EPM, TST, and fear conditioning showed no obvious differences between WT and TG mice (Fig. 6G–L and Supplementary Figs. 1A–G). Intriguingly, we found that the grasping strength and motor coordination were impaired, and pole-climbing time was increased, in TG mice compared to WT mice (Fig. 6M–O).

### Transcriptome analysis of epileptic variant KI mice

**Fig. 7:**
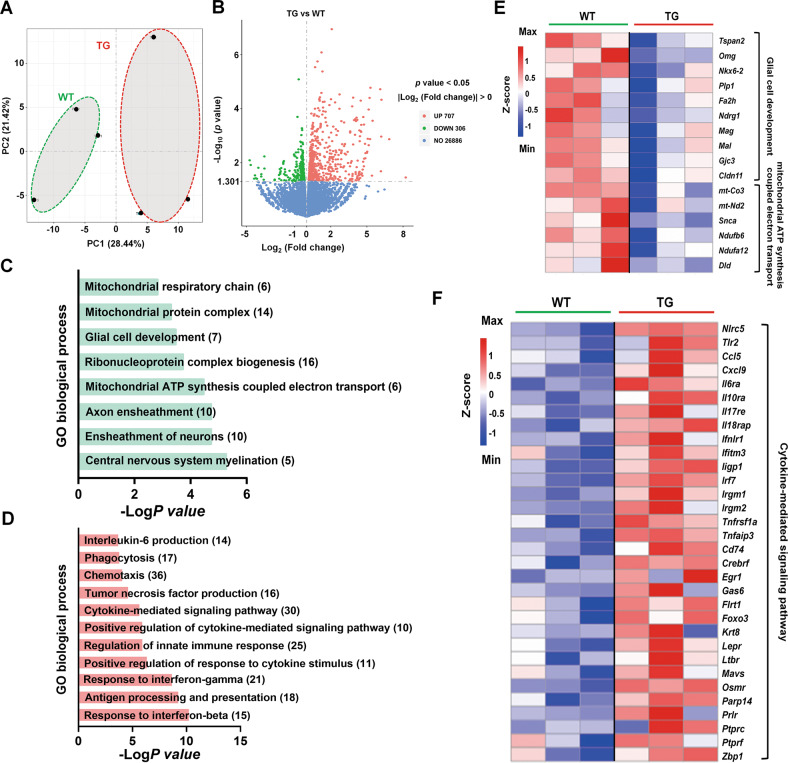
Transcriptome analysis of G82R/L85P variant knock-in mice. **A** PCA score plots revealed a distinct separation between components in WT and TG mice. **B** DEGs between WT and TG mice are shown in a volcano plot. **C**, **D** The representative GO pathways enriched by down- and up-regulated DEGs between WT and TG mice are shown. **E** Downregulated DEGs enriched in “Glial cell development” and “Mitochondrial ATP synthesis coupled electron transport” pathways are shown. **F** Upregulated DEGs enriched in the “Cytokine-mediated signaling pathway” are shown.

We then performed RNA-seq to investigate the underlying mechanism of G82R/L85P variant TG mice. The principal components analysis (PCA) score plots revealed a distinct separation of components between WT and TG mice (Fig. 7A), and the volcano plot showed DEGs between WT and TG mice (Fig. 7B). The gene and FPKM expressions, Pearson correlation, and Venn diagram are presented in Supplementary Figs. 2 and 3. We present the GO pathways enriched by the decreased and increased DEGs in Fig. 7C, D. Notably, we found that the decreased DEGs were enriched in “Central nervous system myelination,” “Ensheathment of neurons,” “Axon ensheathment,” and “Glial cell development,” which were related to the function of astrocytes in the CNS (Fig. 7C). The upregulated DEGs were enriched in neuroinflammation-associated signaling pathways, such as “Response to interferon-beta,” “Response to interferon-gamma,” “Regulation of innate immune response,” and “Cytokine-mediated signaling pathway” (Fig. 7D). We listed the representative decreased DEGs enriched in “Glial cell development” and “Mitochondrial ATP synthesis coupled electron transport” pathways, and increased DEGs enriched in “Cytokine-mediated signaling pathway” (Fig. 7E, F).

**Fig. 8:**
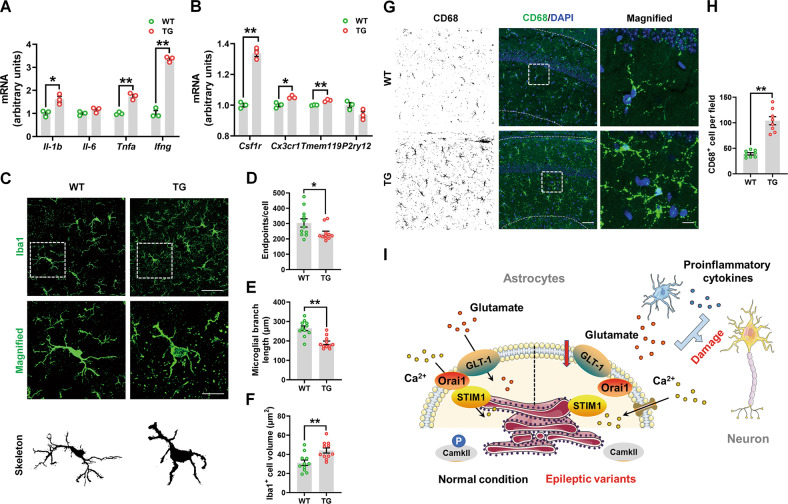
G82R/L85P variant knock-in mice had neuroinflammation. **A**, **B** The mRNA expression levels of *Il-1b*, *Il-6*, *Tnfa*, *Ifng*, *Csf1r*, *Cx3cr1*, *Tmem119*, and *P2ry12* were determined by qRT-PCR. **C** Immunofluorescence staining of Iba1-positive cells in the hippocampus of WT and TG mice. Scale bar, 20 μm. Magnified Iba1-positive cells are shown in the middle column of panel (**C**), and their skeletal images are shown in the bottom column of panel (**C**). Scale bar, 5 μm. **D**–**F** Quantification of endpoints per cell, branch length, and the volume of Iba1-positive cells in panel (**C**). *n* = 11 per group. **G** Immunofluorescence staining of CD68-positive cells in the hippocampus of WT and TG mice. Scale bar, 50 μm. Magnified CD68-positive cells are shown in the right column of panel (**G**). Scale bar, 10 μm. **H** Quantification of the numbers of CD68-positive cells in panel (**G**). *n* = 8 per group. **I** Schematic model of the study. In the normal condition, GLT-1 is responsible for glutamate uptake and interacts with STIM1/Orai1 to maintain the Ca^2+^ refilling and phosphorylation of CaMKII in the ER. However, epileptic variants (G82R, L85P, and P289R) decrease the expression and function of GLT-1 and inhibit STIM1/Orai1-mediated SOCE together with the phosphorylation of CaMKII. Glutamate excitotoxicity and microglia-released proinflammatory cytokines damage the hippocampal neurons and induce epilepsy. Results are expressed as mean ± SD. ^**^P < 0.01, ^**^P < 0.05 vs. WT. Statistical significance was determined using Student’s *t* test.

Because the upregulated DEGs were enriched in the neuroinflammatory pathways, and glutamate excitotoxicity is a crucial cause of neuroinflammation in epilepsy [52], we examined the inflammatory markers in WT and TG mice. We found that the mRNA expression of proinflammatory cytokines, such as IL-1β, TNF-α, and IFN-γ, and microglial homeostatic markers (CSF1R, CX3CR1, and TMEM119) were increased in the hippocampus of TG mice compared to the WT mice (Fig. 8A, B). The immunofluorescence staining demonstrated reductions in microglial endpoints and process complexity, an increased microglial volume, and elevated CD68 (an activation marker of microglia) positive cells in the hippocampus of TG mice compared with WT mice (Fig. 8C–H), indicating that microglia change from a “resting” ramified phenotype to an “activated” bushy phenotype.

## Discussion

The glutamate transporter GLT-1 plays a key role in terminating glutamate excitotoxicity in the synaptic cleft, and thus its dysfunction contributes to neurodegenerative diseases, such as PD, AD, and amyotrophic lateral sclerosis (ALS) [38, 40, 53, 54], and neurological diseases, such as epilepsy and stroke [17, 18, 55]. Previously, three *SLC1A2* variants, Gly82Arg, Leu85Pro, and Pro289Arg, were reported to be clinically linked with epilepsy [17, 18]. In this study, we further verified that these variants reduced the expression and function of GLT-1, which may be caused by the regulation of STIM1/Orai1-mediated SOCE. Additionally, we established *SLC1A2* variant KI mice to provide a basis for the role of GLT-1 in epilepsy (Fig. 8I).

Of the above variants, Gly82 and Leu85 were located in TM2 of glutamate transporters. As mentioned above, it is widely believed that the trimeric interface of the glutamate transporters, which involves TM2, 4, and 5, remains rigid during the transport process [41]. However, several groups, including ours, have revealed that several amino acid residues, including Leu85 in TM2, are essential for the transport behavior and anion channels [36, 51, 56]. In this study, we found that G82R and L85P significantly reduced the expression and function of GLT-1 and glutamate-elicited currents. In addition, the G82R/L85P mutant KI mice manifest decreased GLT-1 expression. These data further support the critical role of TM2 in the transport process. The effects of G82R and L85P on the glutamate transporter are explained by their role in determining the GLT-1 location in the membrane because maintaining the side-chain structures of the glutamate transporter did not abolish the effects of variants. Because the G82R and L85P variants significantly suppressed the expression and function of GLT-1 (nearly 90%), it was difficult to evaluate the relative motion between TM2 and TM5.

SOCE is the main calcium regulator for non-excitatory neural cells, such as astrocytes and microglia [47, 48] and astrocytic knockdown of GLT-1 impaired the calcium signaling pathway [39]. Both STIM1 and STIM2 were found to be elevated in the dentate gyrus, CA1 and CA3 regions of chronic epileptic mice and in a hippocampal sample from a patient with medial temporal lobe epilepsy [57]. Pharmacologic inhibition of SOCE suppresses the interictal spikes and epileptic burst activity [57]. Thus, STIM1/Orai1 mediated SOCE could modulate neuronal excitation and regulate synaptic plasticity [58]. In this study, we evaluated the effects of epileptic variants on SOCE. First, we found that GLT-1 may be a partner of the SOCE machinery, as evidenced by the interaction between GLT-1 and STIM1/Orai1. In addition, the disease-related mutants decreased the Orai1 expression and reduced the interaction between GLT-1 and STIM1/Orai1, suggesting that they may inhibit SOCE activity. Previously, suppression of SOCE was reported to inhibit the rhythmized epileptic burst activity in chronic epileptic hippocampal slices [57]. Besides, overexpression of Orai1 in the mice brain enhances the Ca^2+^ response and neuronal activity, and leads to spontaneous occurrence of seizure-like events [59, 60]. We concluded that the contradictory results may be due to the effects of SOCE on different cell types, considering that we focused on SOCE in astrocytes in the present study. In addition, we hypothesized that epileptic variants possibly decrease the SOCE activity in astrocytes due to their compensatory effects. In the normal condition, exhausted ER Ca^2+^ level triggers STIM to interact with Orai1 and TRP families to activate the refilling of ER Ca^2+^ through SOCE [49]. Epileptic variants significantly reduced the expression and function of GLT-1, which induced extracellular accumulation of glutamate. Since glutamate excitotoxicity affects calcium homeostasis in astrocytes [61, 62], SOCE inhibition in epileptic variants may be resistant to these effects.

Inspired by the emerging role of TM2 in glutamate transport and our in vitro results, we established the *SLC1A2* variant (G82R/L85P) KI mice. The mice manifested decreased GLT-1, Orai1, and phosphorylation of CaMKII expression, which is consistent with our in vitro results that GLT-1 deficiency may interrupt SOCE activity. In addition, the hyperactive behavior of mice may be due to glutamate excitotoxicity-evoked neuronal activity. We also found impaired movements in the TG mice, which is in line with previous studies that showed that reduced astrocytic GLT-1 may induce a parkinsonian phenotype [39, 63, 64]. Using RNA-seq, we identified that the decreased DEGs were enriched in glial cell development in TG mice. *Tetraspanin-2* (*Tspan2)*, *Proteolipid protein 1* (*Plp1*), *Nkx6-2*, *Fatty acid 2-hydroxylase* (*Fa2h*), *N-myc downstream regulated gene 1* (*Ndrg1*), and *Claudin11* (*Cldn11*) participate in glial development and maintenance of the myelin sheath, and their dysfunction is related to spastic ataxia and hypomyelination [65–69]. *Tspan2* and *Plp1* are well-characterized members of the tetraspanin superfamily, *Tspan2* contributes to the early stages of the oligodendrocyte differentiation into myelin-forming glia, and *Plp1* is essential for the axon-supportive function of myelin [70, 71]. Both *Plp1* and *Tspan2* localize to compact myelin, while *Plp1* is also responsible for the biogenesis and structure of myelin, and the maintenance of axonal integrity [72]. *Nkx6-2* is involved in oligodendrocyte maturation and its mutations leads to the hypomyelination and spastic ataxia phenotype in humans [66]. *Fa2h* is responsible for the formation of 2-hydroxy galactolipids in nervous system myelin, and its mutations cause leukodystrophy and spastic paraplegia [67, 73]. *Ndrg1* is indispensable for the maintenance of the myelin sheath, and its deficiency leads to Schwann cell dysfunction and progressive demyelinating disorder [68, 74]. *Cldn11*, a claudin family of tight junction proteins, is a major component of nervous system myelin and it also regulates the proliferation and migration of oligodendrocytes [75]. Mice lacking *Cldn11* exhibit central auditory deficits and reduced anxiety-like behavior [76]. The increased DEGs were enriched in the neuroinflammation-related pathways, which was confirmed by examining the cytokine expression and microglial morphology. A previous study reported that glutamate excitotoxicity activates microglia to induce neuroinflammation in epilepsy [52]. However, we did not observe spontaneous epileptic seizures in these mice. This may be because of the high embryo mortality in GLT-1 deficiency, as reported previously [42], which is why we were unable to obtain an adequate number of mutant mice. In addition, specific hippocampal mutant mice may be required to observe seizures.

In the present study, we confirmed that the clinically reported epileptic *SLC1A2* variants (G82R, L85P, and P289R) decreased the expression and function of GLT-1, and inhibit glutamate-elicited currents in vitro. The underlying mechanism possibly involves the regulation of STIM1/Orai1-mediated SOCE machinery by GLT-1. Furthermore, we established *SLC1A2* variant KI mice to provide a basis for the role of GLT-1 in epilepsy. Taken together, glutamate transporters were found to be an emerging therapeutic target for epilepsy intervention.

## Material and methods

### Reagents

Anti-CaMKII (#4436), Phospho-CaMKII (Thr286) (#12716), STIM1 (#5668), and Integrin β1 (#34971) antibodies were purchased from Cell Signaling Technology (Danvers, MA, USA). Anti-GLT-1 (sc-365634) antibodies were purchased from Santa Cruz Biotechnology (Dallas, TX, USA). Anti-Orai1 (66223-1-Ig) and Actin (23660-1-AP) antibodies were purchased from Proteintech Group (Rosemont, IL, USA). DyLight 488 goat anti-mouse IgG (H+L) (70-GAM4882) and DyLight 594 goat anti-rabbit IgG (H+L) (70-GAR5942) were purchased from Multi Sciences (Hangzhou, China). Horseradish peroxidase (HRP)-labeled goat anti-rabbit IgG and HRP-labeled goat anti-mouse IgG were purchased from Beyotime Biotechnology (Shanghai, China). D-[^3^H]-Asp was purchased from PerkinElmer (Waltham, Massachusetts, USA). EZ-Link Sulfo-NHS-SS-Biotin was purchased from Thermo Scientific (#21331, Waltham, MA, USA).

### Generation and subcloning of mutants

The cysteine-less EAAT2 (coded by *SLC1A2*, Gene ID: 20511) was constructed in the vector pBluescript SK (Stratagene, La Jolla, CA, USA), and served as a template for site-directed mutagenesis. Mutant glutamate transporters were generated using QuikChange Site-Directed Mutagenesis Kit (Toyobo, Osaka, Japan), and ascertained using full-length sequencing (Sangon Biotech, Shanghai, China) according to our previous study [35, 51]. The pBluescript SK plasmid was used as the negative control.

### Cell culture and transient transfections

HeLa cells were purchased from ATCC (Manassas, VA, USA) and cultured in DMEM basic medium (Invitrogen, Carlsbad, CA, USA) with 10% fetal calf serum (Invitrogen, Carlsbad, CA, USA), 100 U/mL penicillin, and 0.1 mg/ml streptomycin (Beyotime Biotechnology, Shanghai, China). Transfections were performed using Lipofectamine™ 3000 Transfection Reagent (#L3000150, Invitrogen, Carlsbad, CA, USA) according to the manufacturer’s protocol. Briefly, cells were seeded to achieve 70–90% confluence before transfection, and the plasmid DNA-lipid complexes were prepared according to the manufacturer’s instructions. Then, the DNA-lipid complexes were added to the cells for 24 h. Subsequently, glutamate transport and cell surface biotinylation were performed.

### Transport assay

Glutamate transport assay was performed according to our previous study [34, 35]. HeLa cells in 24 plates transfected with different mutants were washed with 1 mL of choline chloride (ChCl) solution (150 mM ChCl, 5 mM KPi, pH 7.4, 0.5 mM MgSO_4_, and 0.3 mM CaCl_2_) for two times. Then, NaCl solution (150 mM NaCl, 5 mM KPi, pH 7.4, 0.5 mM MgSO_4_, and 0.3 mM CaCl_2_) containing 0.4 mCi (0.15 mM) D-[^3^H]-aspartate (PerkinElmer, Waltham, MA, USA) was added, and the solution was incubated for 10 min at room temperature. Next, 1% SDS was added after washing the cells twice with NaCl solution, and the D-[^3^H]-aspartate radioactivity was detected. Because both glutamate and aspartate are the substrates for the high-affinity glutamate transport system [77, 78], the ^3^H-labeled aspartate could reflect the transport function for glutamate.

### Cell surface biotinylation

Cells in six-well plates were washed with 0.01 M PBS and incubated with 0.5 mg of EZ-Link Sulfo-NHS-SS-Biotin (#21331, ThermoFisher Scientific, Waltham, MA, USA) on ice for 40 min. Then, cells were incubated with 100 mM glycine on ice for 20 min. The total protein was extracted using IP lysis buffer (Beyotime Biotechnology, Shanghai, China). Beads were added to the total protein and incubated at 4 °C for 1 h. After centrifugation, the supernatant included the non-biotinylated protein. The beads were washed with 0.01 M PBS and incubated with the loading buffer at 60 °C for 30 min. After centrifugation, the supernatant containing biotinylated protein was collected.

### Co-immunoprecipitation assay (Co-IP)

Co-IP was performed using the Immunoprecipitation Kit (#PK10008, Proteintech, Rosemont, IL, USA) according to the manufacturer’s instructions. Briefly, protein samples were extracted using IP Lysis buffer supplemented with protease inhibitor. Protein samples were quantified by the BCA protein assay kit. A total of 2 μg of specific capture antibody and incubation buffer were added to 2 mg of protein samples and incubated at 4 °C for 24 h. Then, 50 μL of protein A/G beads were added to spin columns to capture the antigen-antibody complex. The antigen-antibody complex was washed with washing buffer and separated using 80 μl of elution buffer. Then, 10 μL of alkali neutralization buffer and 23 μL of 5 × loading buffer were added to the complex and analyzed by Western blot.

### Western blot

Cells and brain tissue were lysed using RIPA buffer supplemented with a protease inhibitor cocktail (Beyotime Biotechnology). Protein samples were quantified using a BCA protein assay kit and separated on 4–20% SDS-PAGE gel. Then, proteins were transferred onto polyvinylidene fluoride (PVDF) membranes and blocked with 5% BSA. The membranes were probed with primary antibodies and on the second day with the HRP-labeled secondary antibody. Images were captured using the GeneGnome XRQ Chemiluminescence imaging system (Gene Company, Hong Kong, China). Protein levels were normalized against that of actin. Quantification of the bands was performed using Image J software. Full and uncropped western blots were provided in Supplemental Material.

### HEK293 cell culture and transfection

HEK293 cells (ATCC, Manassas, VA, USA) were cultured in DMEM basic medium (Invitrogen, Carlsbad, CA, USA) with 10% fetal calf serum (Invitrogen, Carlsbad, CA, USA), 100 U/ml penicillin, and 0.1 mg/ml streptomycin (Beyotime Biotechnology, Shanghai, China) at 37 °C in a 5% CO_2_ incubator. The day before patch-clamp recording, HEK293 cells were transiently transfected with SK, wild type (WT), or mutants (82R, L85P and P289R) using Lipo6000.

### Patch-clamp recording

Whole-cell recording was performed according to our recent study [51]. Pipettes were fabricated from borosilicate glass (World Precision Instruments, Sarasota, FL, USA) using a micropipette puller (PC-10, Narishige, Japan). Currents were elicited in HEK293 cells by 2 s of voltage ramps at −100 to +40 mV and a frequency of 0.1 Hz with a holding potential of −20 mV. Currents were amplified using Axopatch 700 A and digitized using Digidata 1440 A (Molecular Devices, San Jose, CA, USA). The standard extracellular solution contained (in mM) 137 NaCl, 4 KCl, 1.8 CaCl_2_, 1 MgCl_2_, 10 HEPES, and 10 glucose, at a pH of 7.4 (with NaOH). The standard intracellular solution contained (in mM) 130 CsCl, 5 MgCl_2_, 5 EGTA, and 10 HEPES, at a pH of 7.2 (with CsOH). For patch recording of glutamate-mediated currents, 20 mM glutamate was added to the extracellular solution.

### Transmission electron microscopy (TEM)

TEM was performed as described previously [79]. HeLa cells transfected with different mutants were fixed using 1% osmium tetroxide plus potassium ferrocyanide 1% in 0.1 M sodium cacodylate buffer and were dehydrated in a graded ethanol series. Afterwards, they were embedded in epoxy resin (SPI-Pon 812 Epoxy Resin Monomer; SPI, Shanxi, China). Ultrathin sections (60–70 nm) were obtained using an ultra-thin microtome (Leica EM UC7, Germany). Images were captured and analyzed using TEM (HT7700; Hitachi, Tokyo, Japan).

### Generation of *SLC1A2* variant mice

*SLC1A2* mutation mice were generated using a CRISPR/Cas9-based approach. The p. Gly82Arg and p. Leu85Pro mutations were introduced into exon 3 under the genetic background of C57BL/6N. Briefly, two sgRNAs were designed using the CRISPR design tool (https://wge.stemcell.sanger.ac.uk//) to target either a region upstream or downstream of exon 3. Then, the on-target activity was screened using a Universal CRISPR Activity Assay (UCATM, Biocytogen Pharmaceuticals Co., Ltd, Beijing, China). To minimize random integrations, we used a circular donor vector. The gene-targeting vector containing 5′ homologous arm, target fragment (exon3 with *p*. Gly82Arg and *p*. Leu85Pro mutations), and 3′ homologous arm was used as a template to repair the double strand breaks (DSBs) generated by Cas9/sgRNA. T7 promoter sequence was added to the sgRNA template by in vitro PCR amplification. Cas9 mRNA, targeting vector, and the sgRNAs were co-injected into the cytoplasm of one-cell stage fertilized C57BL/6N eggs. The injected zygotes were transferred to the oviducts of Kunming pseudopregnant females to generate F0 mice. F0 mice with the expected genotype confirmed by tail genomic DNA PCR and sequencing were mated with C57BL/6 mice to establish germline-transmitted F1 heterozygous mice. Further, F1 heterozygous mice were genotyped by tail genomic PCR, southern blot, and DNA sequencing. In this study, we obtained four *SLC1A2* variant mice: three males and one female, and their allocation were randomized and blinded. The littermates of wild-type (WT) mice (four males and one female) were used as controls. The mice were housed in standard facilities according to the Institutional Animal Care and Use Committee of Guangzhou Medical University and National Institute of Health guidelines on the care and use of animals (NIH Publications No. 8023, revised 1978).

### Behavioral tests

#### Open field test (OFT)

OFT was performed as described previously [79, 80]. Before the OFT, mice were habituated in the testing room for 1 day. Mice were placed in a box (40 × 40 × 40 cm) and allowed to explore for 15 min. Each mouse was placed individually in the central sector, and its locomotion was recorded using a video tracking system (EthoVisione XT software, Beijing, China). The total distance traveled, movement speed, the time spent in the central and the peripheral zone were recorded during the 15 min test period. After each trial, the apparatus was cleaned with 75% ethanol.

#### Elevated plus maze (EPM)

The EPM consisted of two open arms (50 × 10 cm), two closed arms (30 × 5 × 15 cm), and a central zone (5 × 5 cm). Mice were placed in the central zone for 5 min, and the time spent by mice in the open and closed arms was analyzed using a video tracking system (EthoVisione XT software, Beijing, China).

#### Pole climbing test

The Pole climbing test was performed as stated previously [80, 81]. The mice were placed on the top of a pole with a length of 75 cm and a diameter of 9 cm. The total time taken by mice to reach the pole base was recorded. Mice were trained for 3 days before the test.

#### Grip strength test

The grip strength test was performed as stated previously [80, 81]. Mice were suspended on a horizontal metal wire of l mm diameter and placed 30 cm above the ground for 10 s, using the two front paws to test their grasp strength. The grasping score was recorded as 3, 2, 1, and 0 if mice grasped the wire with two hind paws, mice grasped the wire with one hind paw, mice failed to grasp the wire, and mice fell, respectively.

#### Rotarod test

Rotarod test was performed as stated previously [80, 81]. Mice were trained on the rotarod cylinder for 3 days at a constant speed of 5 rpm. On the test day, the speed of the rotarod cylinder was accelerated from 4 to 40 rpm within 5 min. The latency time to fall of mice was recorded.

#### Tail suspension test (TST)

Mice were fixed in a suspension box at 1 cm from the tip of their tails. The mice heads were about 10 cm above the ground. After adaptation for 2 min, the immobility time of mice was recorded using a video tracking system (EthoVisione XT software, Beijing, China).

#### Fear conditioning test

The fear conditioning test was performed using NIR Video Fear Conditioning Package for Mouse (Med Associates, Vermont, USA). On day 1, after habituation for 180 s, the mice were exposed to a tone (75 dB, 2800 Hz, 30 s) and then to the same tone combined with electrical shock (1 mA) for 1 s, which was repeated four times at an interval of 110 s. On day 2, the mice were placed in the same chamber as on day 1 for 5 min without tone or electrical shock to assess context-dependent fear conditioning. On day 3, mice were placed in a black-colored chamber of the same size to assess the baseline activity for 3 min and then were exposed to the same tone as on day 1 for 3 min to assess tone-dependent fear conditioning. To evaluate the context- and tone-dependent conditioning, freezing scores were obtained by the Video Freeze® Software system (Med Associates, Vermont, USA) and expressed as a percent of baseline activity.

### RNA-sequencing (RNA-seq)

RNA-seq was performed as described previously [39, 79]. Briefly, hippocampal tissues were collected and RNA was extracted using Trizol (Invitrogen, Carlsbad, CA, USA). The cDNA libraries were prepared using the NEBNext® Ultra™ RNA Library Prep Kit for Illumina®, and libraries were sequenced on a HiSeq 2500 instrument platform (San Diego, CA, USA). Sequencing was performed at Novogene Inc. (Beijing, China). Read numbers were calculated using HTSeq v0.6.0, and fragments per kilobase of transcript-per-million mapped reads (FPKM) of each gene were calculated based on the length of the gene and read counts mapped to the analyzed gene. Genes were classified as differentially expressed based on the cutoffs of fold change (FC) > 1, false discovery rate (FDR) < 0.1, and adjusted *P* < 0.05. Gene ontology (GO) pathway analysis of differentially expressed genes (DEGs) was performed using the R package (v 3.5.1). RNA-seq data used in this study are available under GEO: GSE213652.

### Quantitative reverse transcription-polymerase chain reaction (qRT-PCR)

Total RNA was isolated using Trizol (Invitrogen, Carlsbad, CA, USA). A total of 1 μg of RNA was reverse-transcribed using the cDNA Reverse Transcription Kit (QIAGEN, Waltham, MA, USA). Gene expression was assessed using qRT-PCR assays. The relative mRNA expression was normalized to that of the actin gene. Quantitative PCR was performed using the primers listed in Supplementary Table 1. Data are from three separate experiments, with each experiment performed in triplicate.

### Immunofluorescence analysis

Mice brains were fixed in 4% paraformaldehyde and dehydrated in 30% sucrose solution. Brains were frozen in OCT buffer, and 30 mm serial coronal sections were cut using a freezing microtome (Leica, Hamburg, Germany). Then, the sections were blocked with 5% BSA and incubated with Iba1 antibody overnight at 4 °C. On the second day, the sections were incubated with fluorescent-labeled secondary antibody, and images were captured using a confocal microscope (SP8; Leica). Quantitative analysis was performed using the Image-Pro Plus 6.0 photogram analysis system (IPP 6.0, Media Cybernetics, Bethesda, MD, USA).

### Statistical Analysis

Data are presented as mean ± standard deviation of the mean (SD). The electrophysiological data were analyzed using pCLAMP 10 software (Molecular Devices, Union City, CA, USA). The resulting *P* values of RNA-seq data were adjusted using the Benjamini-Hochberg approach for controlling false discovery rates. The Student’s *t*-test was used for comparisons between two groups, and one-way analysis of variance (ANOVA) followed by the Tukey’s post hoc test was used for multiple comparisons. Differences with a *P* value *<* 0.05 were considered statistically significant. The statistical analyses were performed using GraphPad Prism 9.0 (GraphPad Software, La Jolla, CA, USA). *P* values are represented as ^*^P < 0.05 and ^**^P < 0.01.

## Supplementary information

Supplementary Figure Legends

Supplementary Figure 1

Supplementary Figure 2

Supplementary Figure 3

Supplementary Table 1

Supplementary Video 1-WT mice

Supplementary Video 2-TG mice

Reproducibility checklist

Original Data File

## Data Availability

RNA-seq data used in this study are available under GEO: GSE213652. Additional data related to this paper are available from the corresponding author upon reasonable request.
