# Supplementary material for: Functional investigation of SLC1A2 variants associated with epilepsy
Source: Cell Death Dis. 2022 Dec 21;13(12):1063. doi: 10.1038/s41419-022-05457-6 (PMC9772344; doi:10.1038/s41419-022-05457-6)
Supplement: Supplementary file 1 — Supplementary Figure Legends [file 41419_2022_5457_MOESM1_ESM.docx]

**Supplementary Figure Legends**

**Supplementary Figure 1. Behavioral performance of G82R/L85P variant knock-in mice.** (A) The number of entries into the center zone of open field. (B and C) Time spent in the open arm and number of open arm entries in the EPM test. (D) Immobility time in TST. (E and F) Freezing levels of WT and TG mice in the contextual test, before the tone was applied. (G) Freezing levels of WT and TG mice after tone-cued fear conditioning was applied. n = 5 for WT mice (four males and one female) and n = 4 for TG mice (three males and one female). Results are expressed as mean ± SD. Statistical significance was determined using Student’s *t* test.

**Supplementary Figure 2. RNA-seq analysis of hippocampal DEGs in WT and TG mice.** (A) Gene expression distribution of RNA-seq data in the hippocampus of WT and TG mice. (B) FPKM density distribution of RNA-seq data in the hippocampus of WT and TG mice. (C) Pearson correlation between samples. (D) FPKM distribution of RNA-seq data in the hippocampus of WT and TG mice.

**Supplementary Figure 3. DEGs between WT and TG mice.** Venn diagram showing the DEGs between WT and TG mice.
