# Supplementary figures and images for: Functional investigation of SLC1A2 variants associated with epilepsy
Source: Cell Death Dis. 2022 Dec 21;13(12):1063. doi: 10.1038/s41419-022-05457-6 (PMC9772344; doi:10.1038/s41419-022-05457-6)

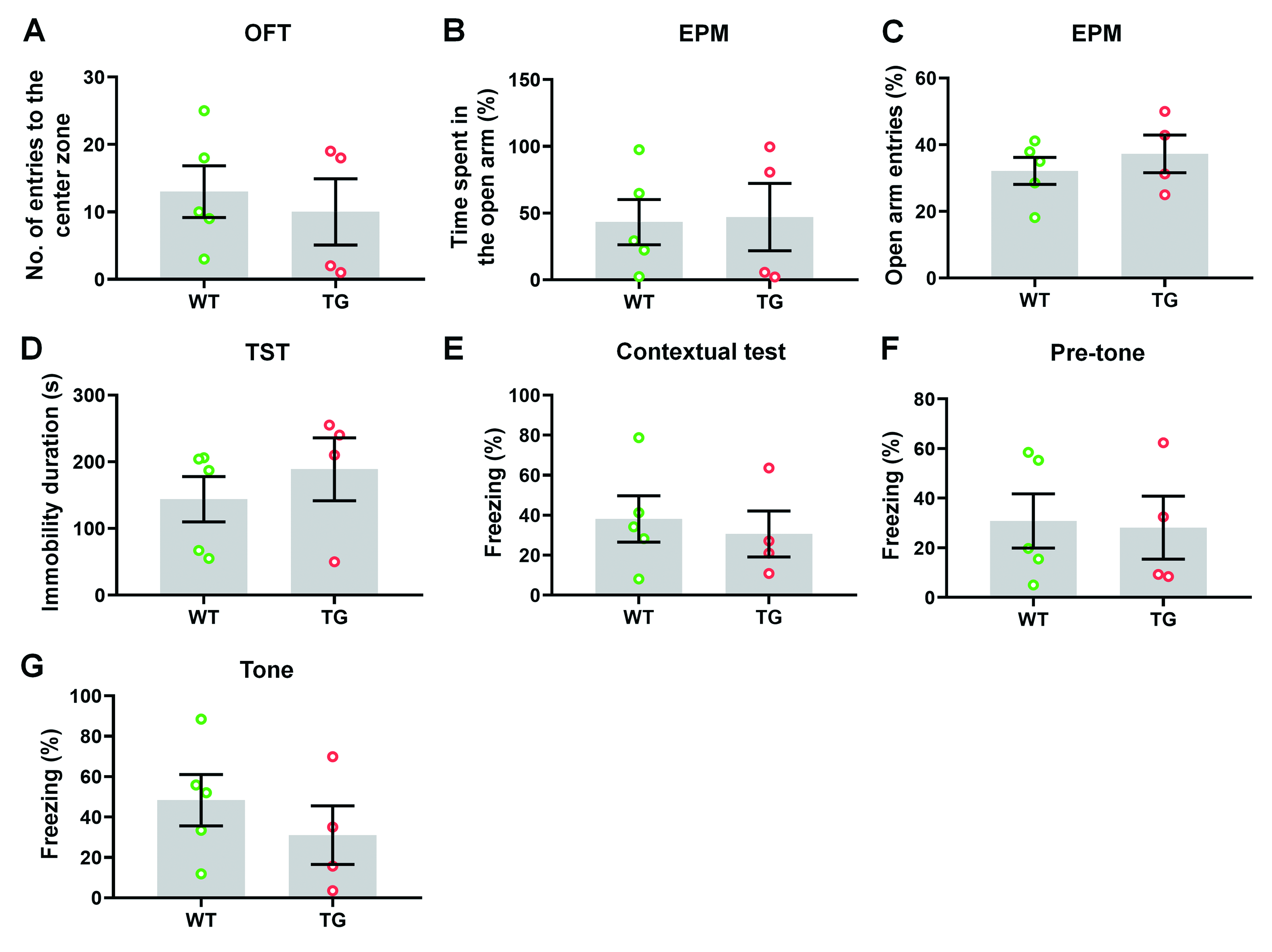

Supplement: Supplementary file 2 — Supplementary Figure 1 [file 41419_2022_5457_MOESM2_ESM.tif]

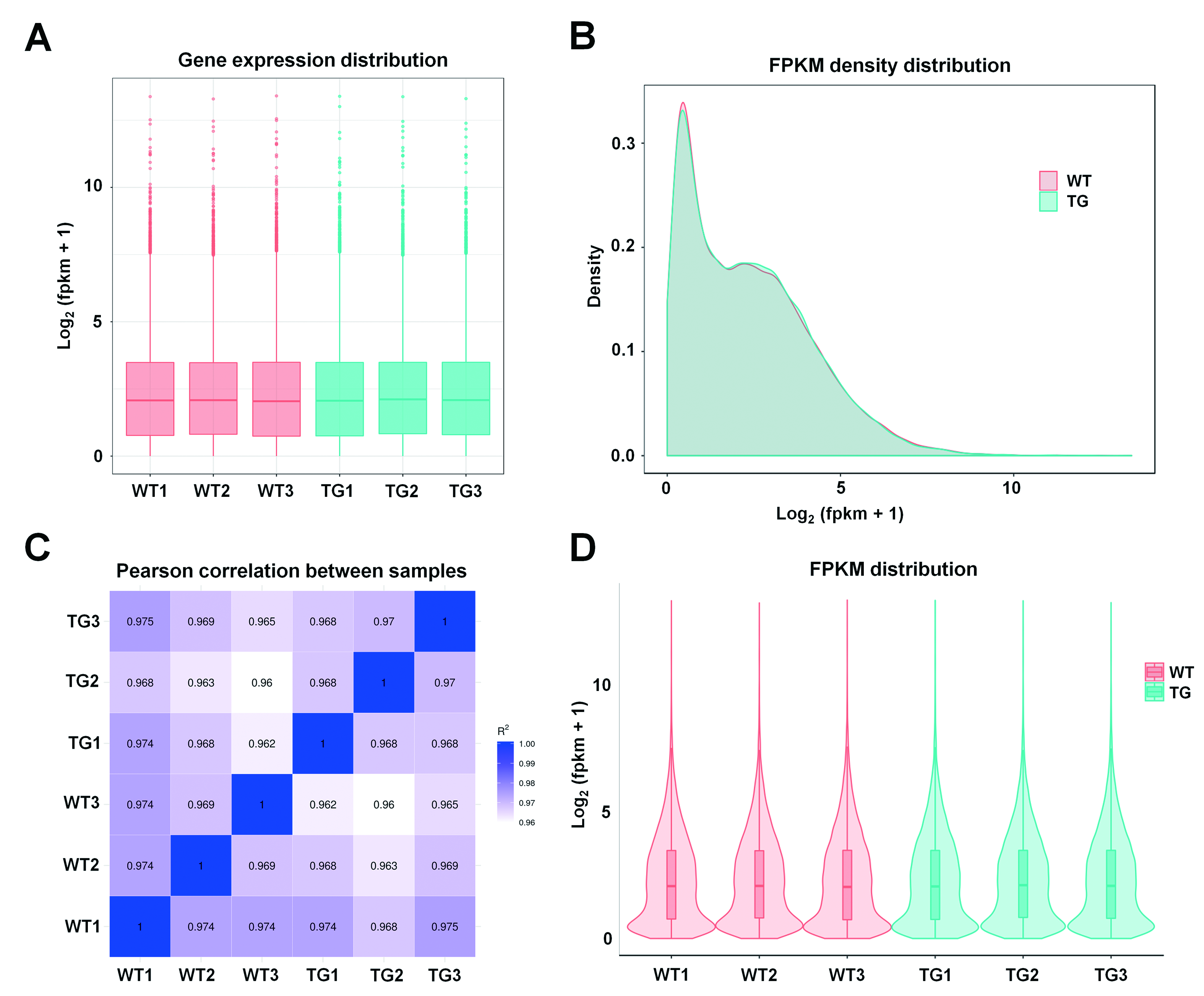

Supplement: Supplementary file 3 — Supplementary Figure 2 [file 41419_2022_5457_MOESM3_ESM.tif]

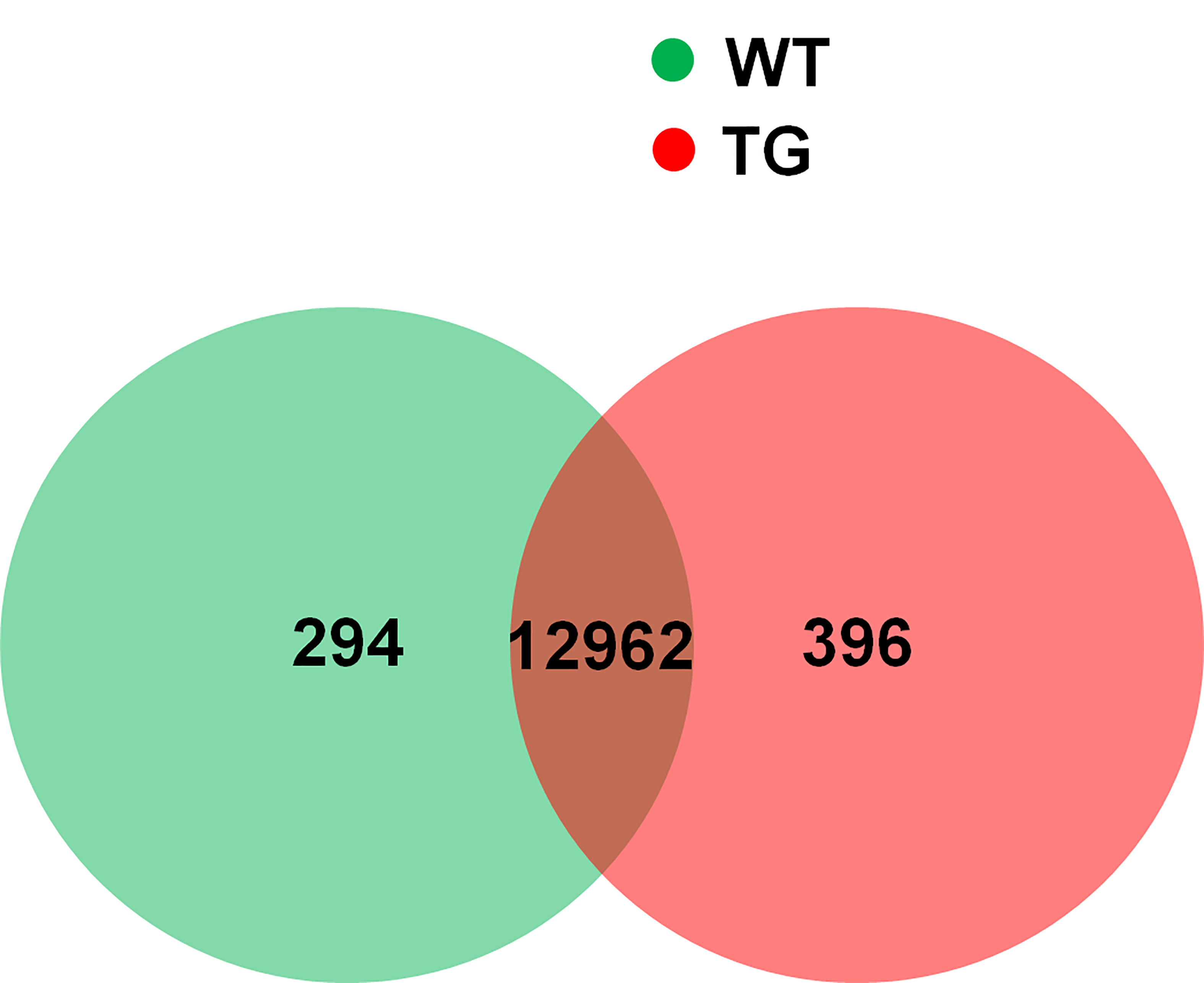

Supplement: Supplementary file 4 — Supplementary Figure 3 [file 41419_2022_5457_MOESM4_ESM.tif]
