# Supplementary material for: Functional investigation of SLC1A2 variants associated with epilepsy
Source: Cell Death Dis. 2022 Dec 21;13(12):1063. doi: 10.1038/s41419-022-05457-6 (PMC9772344; doi:10.1038/s41419-022-05457-6)
Supplement: Supplementary file 5 — Supplementary Table 1 [file 41419_2022_5457_MOESM5_ESM.docx]

**Supplementary Table 1. Primer used for qRT-PCR.**

| Mouse gene | Primer sequence (5′-3′) |
| --- | --- |
| *Il-1b* | F: AATGCCACCTTTTGACAGTGAT  R: TGCTGCGAGATTTGAAGCTG |
| *Il-6* | F: AGGATACCACTCCCAACAGACC  R: AAGTGCATCATCGTTCATACA |
| *Tnfa* | F: CACGTCGTAGCAAACCACC  R: TGAGATCCATGCCGTTGGC |
| *Ifng* | F: TGGCAGGAGATGTCTACACT  R: GAAGCACCAGGTGTCAAGTC |
| *Csf1r* | F: CCTCAAACGTGGAGACACCAA  R: CGTGTGCCAACATCATTGCT |
| *Cx3cr1* | F: CAACCCCTTTATCTACGCCTT  R: GACCCATCTCCCTCGCTTG |
| *Tmem119* | F: CTGACATTCTGGCTGCTACC  R: CACCCTTCACAGGCTTTGCTC |
| *P2ry12* | F: TTTGCTGGGCTCATCACGAAC  R: ACTGAAGTAACTTGGCACACC |
| *Actin* | F: CTACAATGAGCTGCGTGTGGC  R: CAGGTCCAGACGCAGGATGGC |

F, forward primer sequence; R, reverse primer sequence.
