# Supplementary material for: Functional investigation of SLC1A2 variants associated with epilepsy
Source: Cell Death Dis. 2022 Dec 21;13(12):1063. doi: 10.1038/s41419-022-05457-6 (PMC9772344; doi:10.1038/s41419-022-05457-6)

Figure 1D

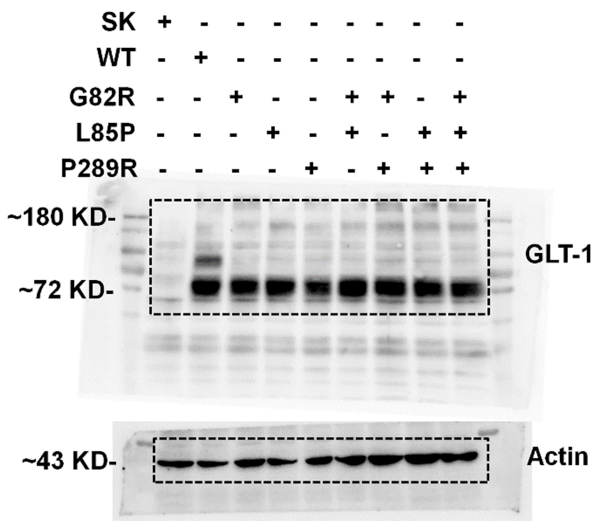

Figure 1F

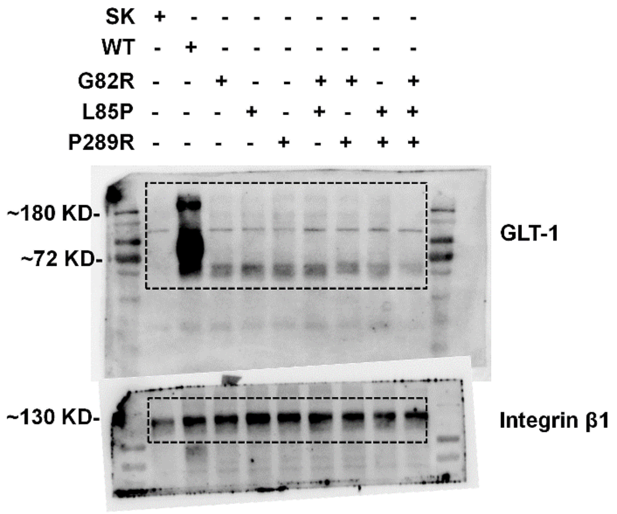

Figure 1H

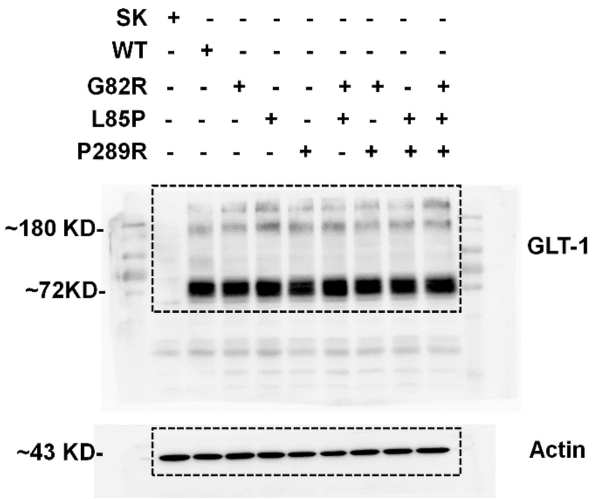

Figure 3C

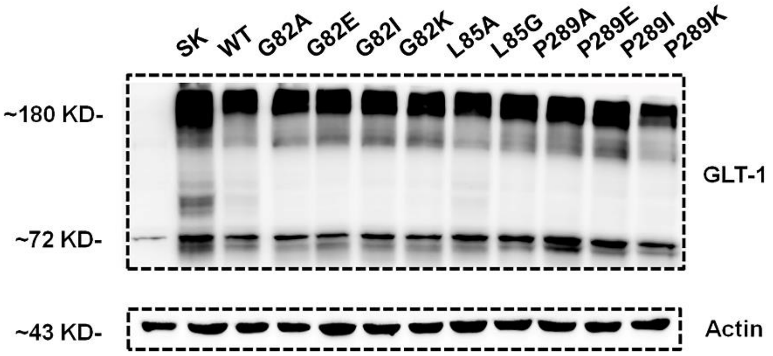

Figure 3E

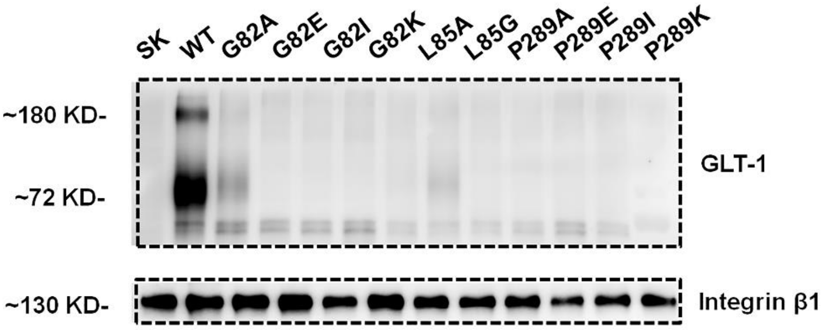

Figure 3G

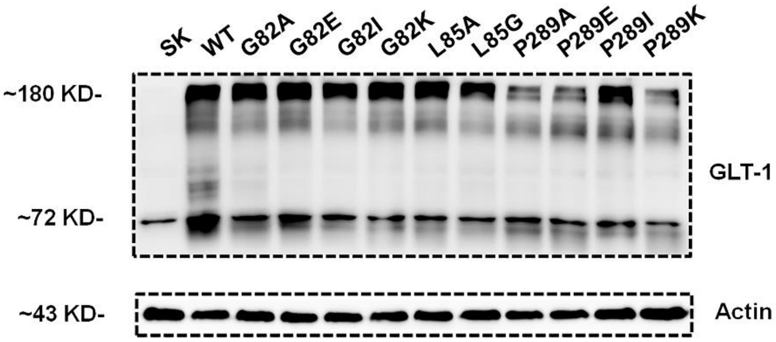

Figure 4A

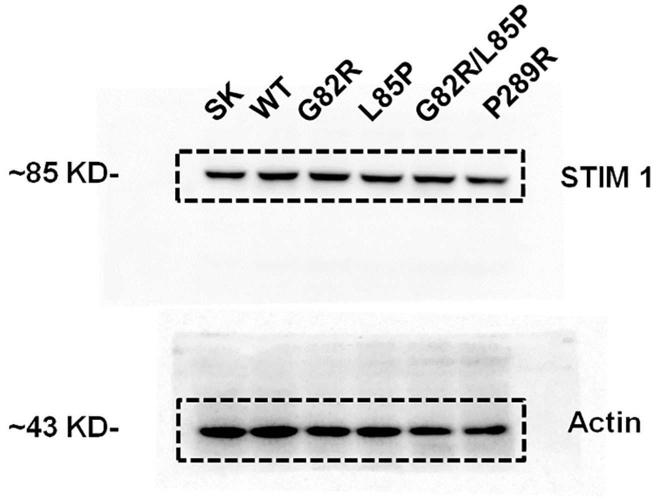

Figure 4C

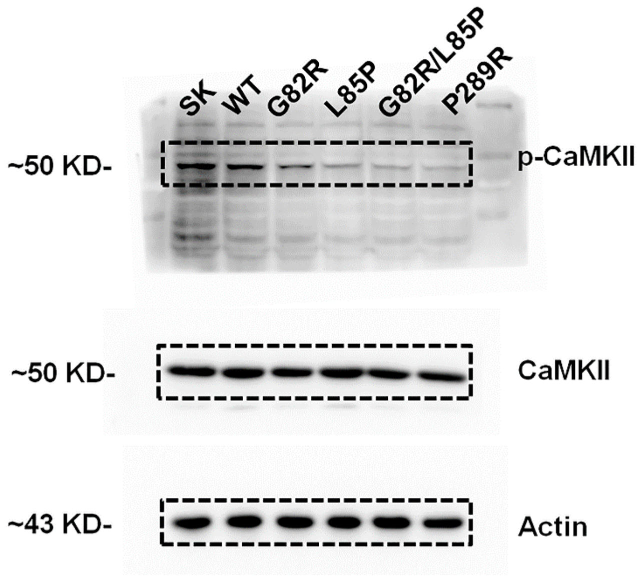

Figure 4E

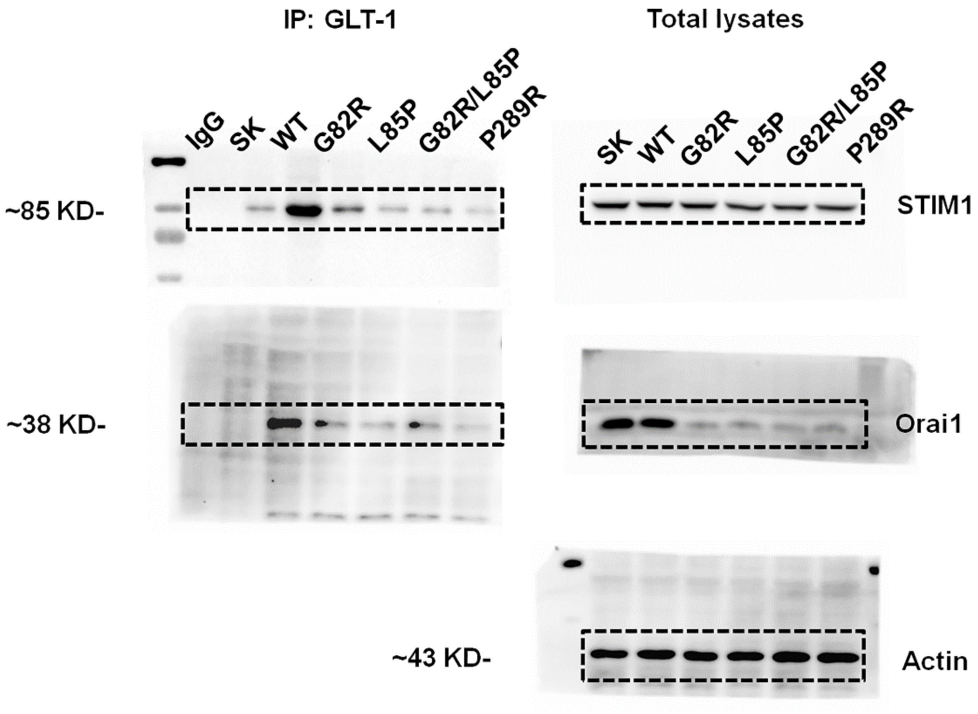

Figure 4G

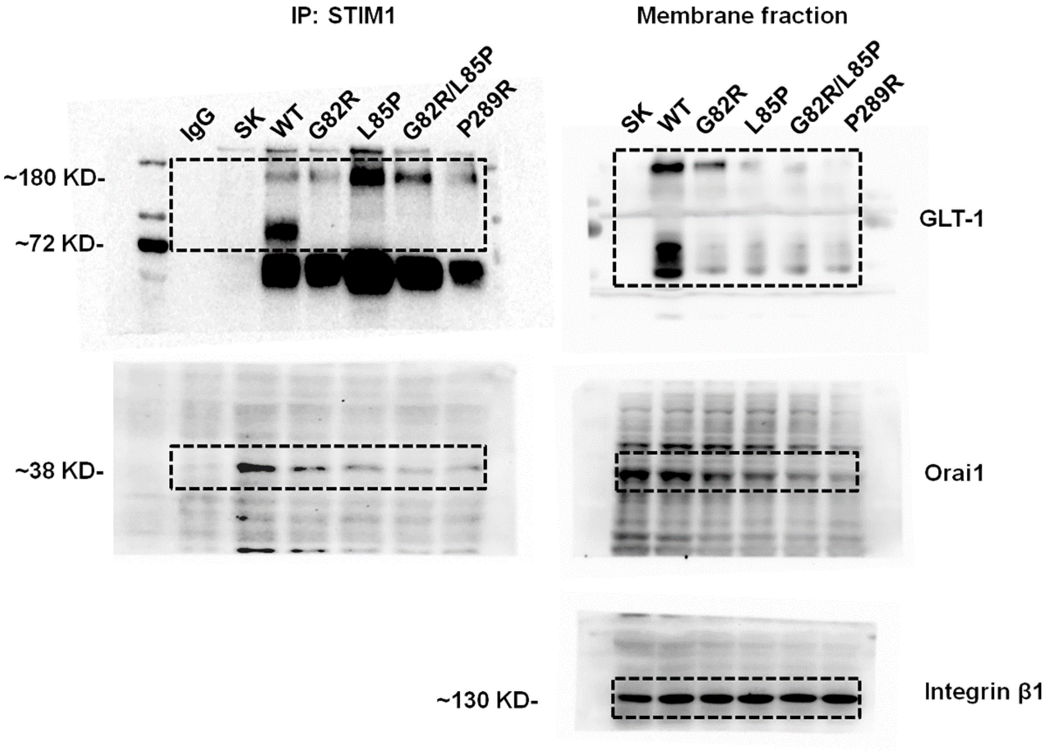

Figure 6C

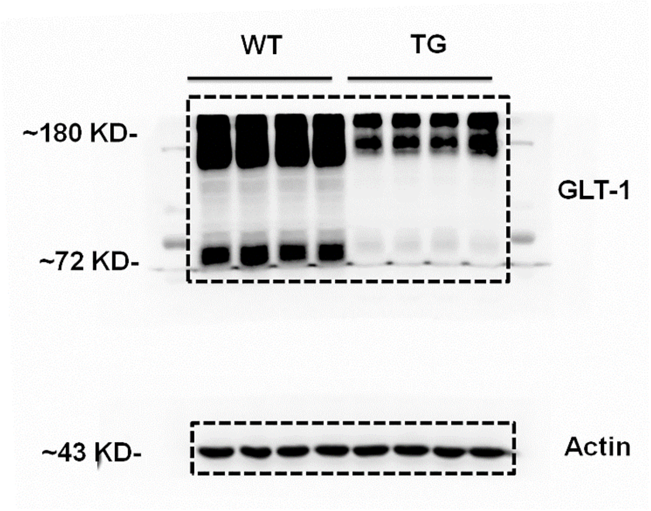

Figure 6E

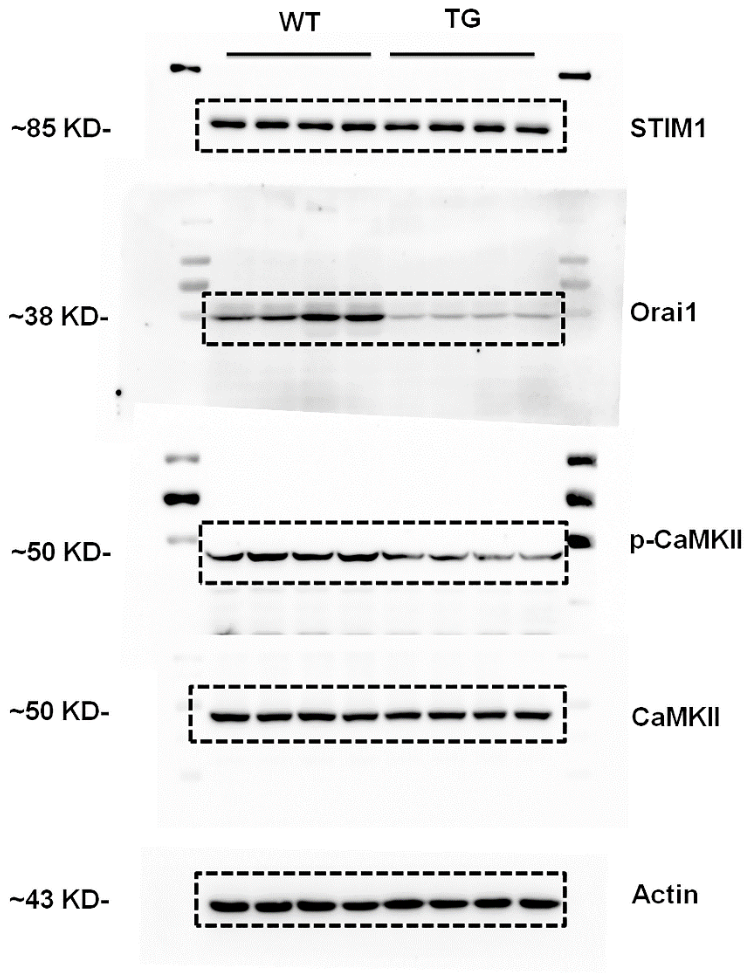

Supplement: Supplementary file 9 — Original Data File [file 41419_2022_5457_MOESM9_ESM.pdf]
